# Cysteine degradation gene *yhaM*, encoding cysteine desulfidase, serves as a genetic engineering target to improve cysteine production in *Escherichia coli*

**DOI:** 10.1186/s13568-017-0389-y

**Published:** 2017-05-10

**Authors:** Gen Nonaka, Kazuhiro Takumi

**Affiliations:** Research Institute for Bioscience Products and Fine Chemicals, Ajinomoto Co., Inc., 1-1 Suzuki-cho, Kawasaki-ku, Kawasaki, Kanagawa 210-8681 Japan

**Keywords:** Cysteine degradation, Cysteine desulfidase, Cysteine desulfhydrase, Cysteine fermentation, *yhaM*

## Abstract

Cysteine is an important amino acid for various industries; however, there is no efficient microbial fermentation-based production method available. Owing to its cytotoxicity, bacterial intracellular levels of cysteine are stringently controlled via several modes of regulation, including cysteine degradation by cysteine desulfhydrases and cysteine desulfidases. In *Escherichia coli*, several metabolic enzymes are known to exhibit cysteine degradative activities, however, their specificity and physiological significance for cysteine detoxification via degradation are unclear. Relaxing the strict regulation of cysteine is crucial for its overproduction; therefore, identifying and modulating the major degradative activity could facilitate the genetic engineering of a cysteine-producing strain. In the present study, we used genetic screening to identify genes that confer cysteine resistance in *E. coli* and we identified *yhaM*, which encodes cysteine desulfidase and decomposes cysteine into hydrogen sulfide, pyruvate, and ammonium. Phenotypic characterization of a *yhaM* mutant via growth under toxic concentrations of cysteine followed by transcriptional analysis of its response to cysteine showed that *yhaM* is cysteine-inducible, and its physiological role is associated with resisting the deleterious effects of cysteine in *E. coli*. In addition, we confirmed the effects of this gene on the fermentative production of cysteine using *E. coli*-based cysteine-producing strains. We propose that *yhaM* encodes the major cysteine-degrading enzyme and it has the most significant role in cysteine detoxification among the numerous enzymes reported in *E. coli*, thereby providing a core target for genetic engineering to improve cysteine production in this bacterium.

## Introduction

Cysteine is an important amino acid for various industries, including pharmaceutical, food, and cosmetics. Bacterial fermentation is the major production method for many amino acids because it combines cost effectiveness and scalability. However, cysteine is one of the few amino acids for which efficient fermentation-based methods are not available, which is a major challenge for the amino acid fermentation industry (Wada and Takagi 2006). Cysteine is known to be highly toxic to cells (Hennicke et al. 2013; Sorensen and Pedersen 1991); therefore, cells are equipped with multifaceted and complex regulatory systems to manage its deleterious effects, thus facilitating strict control of the intracellular cysteine levels (Kredich 2008). Stringent metabolic regulatory control and toxicity of cysteine are the main factors that hinder the establishment of an effective production method in bacteria. Understanding the complexity of this regulatory process is crucial for the optimization of metabolic flux toward cysteine to facilitate its over-production.

The mechanisms responsible for regulating cysteine synthesis include feedback inhibition of key metabolic enzymes in the cysteine biosynthetic pathway, i.e., serine acetyltransferase (SAT) and 3-phosphoglycerate dehydrogenase (3-PGDH). These enzymes play major roles in controlling cysteine biosynthesis through feedback inhibition by cysteine and serine, respectively (Al-Rabiee et al. 1996; Kai et al. 2006). A master regulator of sulfur metabolism, CysB, controls the expression of most of the transporter genes involved in the uptake of various sulfur-containing compounds across cell membranes, as well as that of biosynthetic genes responsible for incorporating these sulfur sources into cysteine (Jagura-Burdzy and Kredich 1983; Kredich 1992; Yamazaki et al. 2016). The CysB-mediated induction of sulfur-assimilating genes promotes cysteine synthesis by coordinating carbon and sulfur flow when cysteine is in short supply. The efflux of excess cysteine using a specific cysteine exporter is another mode of regulation. The cysteine-inducible efflux pump encoded by *cefA* in *Pantoea ananatis* is known to be involved in this regulation by serving as a safety valve when cysteine is present in excess (Takumi and Nonaka 2016).

The degradation of cysteine by specific enzymes comprises an additional mode of regulation. Cysteine desulfhydrases (CDs) are among the best-characterized major cysteine-degrading enzymes in *Escherichia coli* and related bacteria (Awano et al. 2003, 2005; Takumi and Nonaka 2016). In addition, cysteine desulfidases are alternative cysteine-degrading enzymes that are less well-characterized as mediators of cysteine regulation compared to CDs (Tchong et al. 2005). Both enzymes catalyze the same reaction where cysteine is decomposed into sulfide, ammonia, and pyruvate. While cysteine desulfidases use a [4Fe-4S] center, CDs use a pyridoxal phosphate center to catalyze the hydrolysis of cysteine to sulfide. In some bacteria, a single major CD plays a central role in cysteine resistance through degradation, including CcdA in *P. ananatis* and its *Salmonella enterica* serovar Typhimurium ortholog CdsH (Oguri et al. 2012; Takumi and Nonaka 2016). These enzymes are cysteine-inducible and they are mediated by a specific transcription regulator, i.e., CcdR in *P. ananatis* and its *S. enterica* ortholog DecR; therefore, it has been proposed that they are involved in a specific mechanism for coping with cysteine toxicity (Oguri et al. 2012; Takumi and Nonaka 2016). In contrast, at least five minor enzymes (CysK, CysM, MetC, TnaA, and MalY) exhibit CD activities in *E. coli* and it has been proposed that they remove excess cysteine (Awano et al. 2005). However, the functional significance of these enzymes in cysteine degradation is controversial because their regulation associated with cysteine is insignificant (Nonaka, unpublished data; see "Discussion"); moreover, they mediate other primary physiological functions via distinctive enzymatic reactions [e.g., CysK and CysM are cysteine synthases; (Becker et al. 1969; Zhao et al. 2006)].

*Escherichia coli* and *P. ananatis* have been extensively studied as hosts for the fermentative production of cysteine (Takumi et al. 2017; Wada and Takagi 2006). Deregulation of the key enzymes related to feedback inhibition is the usual method employed for bacterial amino acid production. Deregulating the two key feedback loops by introducing feedback inhibition-insensitive mutants of 3-PGDH and SAT are the main options for cysteine overproduction (Al-Rabiee et al. 1996; Kai et al. 2006). Enhancing cysteine efflux pumps is also crucial for the downregulation of intracellular cysteine synthesis to bypass any toxic effects as well as for the secretion of cysteine into the fermentation medium to facilitate further isolation processes. Several efflux pumps are known to facilitate cysteine production including YdeD, YfiK, and multidrug exporters in *E. coli*; and CefA and CefB in *P. ananatis* (Dassler et al. 2000; Franke et al. 2003; Takumi and Nonaka 2016; Takumi et al. 2017; Yamada et al. 2006). Management of intracellular degradation activity is also essential for improving production; therefore, CDs and many other degradative enzymes have been identified and studied extensively. In *P. ananatis*, the discovery of *ccdA*, the only major CD encoded by this bacterium, almost fully addressed the problem because deletion of this gene virtually eliminated the cellular degradation of cysteine, thereby drastically increasing its synthesis (Takumi et al. 2017). Similarly, the deletion of CDs in *E. coli* was shown to be effective for increasing the synthesis of cysteine, where the stepwise deletion of known CDs decreased the intracellular CD activity, resulting in increased cysteine synthesis proportional to the number of deleted genes (Awano et al. 2005). However, even after the complete deletion of the five known CDs in *E. coli*, significant hydrogen sulfide-forming cysteine degradation activity levels were detected, which suggests that additional CDs or cysteine desulfidases that presumably have negative effects on cysteine synthesis may need to be identified. Moreover, a cysteine-inducible cysteine degradative activity was observed in the quintuple mutant, which suggests that at least one of the unknown decomposers of cysteine that remain to be identified might be cysteine-responsive (Awano et al. 2005). Identifying the unknown gene that possibly encodes a core cysteine degradation enzyme in *E. coli* will substantially contribute to our physiological understanding of cysteine metabolism and will thus help overcome the challenge of optimizing the fermentative production of cysteine in *E. coli*.

## Materials and methods

### Strains, plasmids, and growth conditions

Bacterial strains and plasmids used in the present study are listed in Table 1. All of the strains were grown at 37 °C in Luria–Bertani (LB) broth, M9 minimal medium (Sambrook and Russell 2001), or fermentation medium (15 g of (NH_4_)_2_SO_4_, 1.5 g of KH_2_PO_4_, 1 g of MgSO_4_·7H_2_O, 0.1 mg of thiamine hydrochloride, 1.7 mg of FeSO_4_·7H_2_O, 0.15 mg of Na_2_MoO_4_·2H_2_O, 0.7 mg of CoCl_2_·6H_2_O, 1.6 mg of MnCl_2_·4H_2_O, 0.3 mg of ZnSO_4_·7H_2_O, 0.25 mg of CuSO_4_·5H_2_O, 0.6 g of tryptone, 0.3 g of yeast extract, 0.6 g of NaCl, 20 g of CaCO_3_, 135 mg of l-histidine HCl·H_2_O, 4 g of Na_2_S_2_O_3_, 2 mg of pyridoxine hydrochloride, and 40 g of glucose per liter). The medium was supplemented with 20 μg mL^−1^ kanamycin and/or 25 μg mL^−1^ chloramphenicol as required.

**Table 1:** Strains and plasmids

Strains and plasmids	Description^a^	Source or reference
Strains		
MG1655	Wild-type *E. col*i MG1655 (ATCC 47076)	ATCC
MG1655Δ*yhaM*	MG1655Δ*yhaM*	This study
Plasmids		
pSTV29	Cloning vector, p15a ori, Cm^r^	Takara
pSTV-ydeD5	pSTV29; *ydeD* (*E. coli*) gene containing upstream 300 bp and 200 bp downstream	This study
pSTV-yhaM7	pSTV29; *yhaM* (*E. coli*) gene containing upstream 300 and 200 bp downstream	This study
pACYC177	Cloning vector, p15a ori, Km^r^	Nippon Gene
pACYC-yhaM1	pACYC177; *yhaM* (*E. coli*) gene containing upstream 300 and 200 bp downstream	This study
pMIV-cysE5	*cysE5* (*E. coli*) under the control of the *ompC* promoter, p15a ori, Cm^r^	(Nonaka and Takumi 2011)

*ATCC* American Type Culture Collection

^a^Cm^r^, Km^r^, resistance to chloramphenicol and kanamycin

Gene deletion was performed in *E. coli* using the λ-Red recombination system developed by Datsenko and Wanner (Datsenko and Wanner 2000). Plasmid pKD46, which carries an arabinose-inducible λ-Red gene, was used to facilitate Red recombination and pMW-λ*attL*-Km^R^-λ*attR* plasmids (Van Dien et al. 2010) were used as templates to obtain PCR-generated gene disruption cassettes for ::*kan*. The primers listed in Table 2 were used to generate these cassettes. To excise markers from the cassettes flanked by *attL*/*R*, we used the phage λ site-specific Int/Xis system (Van Dien et al. 2010), where we employed the pMW-int-xis plasmid encoding the Int/Xis recombinase.

**Table 2:** Oligonucleotides

Primer name	Sequence (5′–3′)	Use
DyhaM-FW	ATGTTTGATTCGACTTTAAATCCGTTATGGCAGCGTTACATCCTCGCCGTTGAAGCCTGCTTTTTTATACTAAGTTGGCA	Construction of Δ*yhaM*
DyhaM-RV	TTATCTGGCCTTGCTCGCCATAATCTCGATAATCTGCCGATCCGTTTGCTCGCTCAAGTTAGTATAAAAAAGCTGAACGA	Construction of Δ*yhaM*
ECOydeD-F	CGCGGATCCAATGGTCATAAATGGCAGCGTAGCGC	Cloning of *ydeD*
ECOydeD-R	CGCGGATCCGCAGGGCGTTGCGGAACAAAC	Cloning of *ydeD*
ECOyhaM-F	CGCGGATCCAAGATGCCTGCCGAGAAGATTAACG	Cloning of *yhaM*
ECOyhaM-R	CGCGGATCCGAGCGAGCTGGAAGCTATCG	Cloning of *yhaM*
yhaM(Ec)RT-F	CTCGATTCCGCGAAGCTAAA	Real-time PCR: detection of *yhaM*
yhaM(Ec)RT-R	CCCCCACTTACCGCTCAA	Real-time PCR: detection of *yhaM*
yhaO(Ec)RT-F	GCCATTATTACGCTGCCGTTT	Real-time PCR: detection of *yhaO*
yhaO(Ec)RT-R	CCATCGGACTTAACGTCTGGAT	Real-time PCR: detection of *yhaO*
metC(Ec)RT-F	AAGCCGCCACCAAATATCTG	Real-time PCR: detection of *metC*
metC(Ec)RT-R	ACACGGCAGTGCCAATCA	Real-time PCR: detection of *metC*

To construct the *yhaM* and *ydeD* expression plasmids, multicopy cloning vectors pSTV29 (Takara, Tokyo, Japan) and pACYC177 (Nippon Gene, Tokyo, Japan) were used as backbones. The PCR-generated fragments of *ydeD* and *yhaM* were digested using *Bam*HI (these sites were designed based on the 5′ and 3′ ends of the primers) and cloned into the corresponding sites in pSTV29 to yield pSTV-ydeD5 and pSTV-yhaM7, and in pACYC177 to yield pACYC-yhaM1. The primers used for plasmid construction are listed in Table 2.

### Screening for genes that confer cysteine resistance

Genomic DNA from *E. coli* wild-type strain MG1655 was digested partially with *Sau*3AI and fragments measuring approximately 10 kb were cloned into the *Bam*HI site of pSTV29 (Takara Bio, Shiga, Japan). The genomic library obtained was introduced into MG1655 by electroporation and the cells were then challenged on M9 plates containing 2–4 mM cysteine for 2 days. Colonies were isolated and candidate genes in the plasmids that conferred cysteine resistance were analyzed by sequencing.

### Growth assay

For the growth assay on plates, overnight cultures produced on regular M9 minimal medium plates (containing MgSO_4_ as a sulfur source) were streaked onto fresh M9 sulfur-free plates (MgSO_4_ was replaced with MgCl_2_) supplemented with 4 mM cysteine and then incubated at 37 °C for 2 days before observing their growth.

For the growth assay in liquid culture, each overnight culture cultivated in a test tube containing 3 mL of M9 minimal medium was diluted 1:100 with fresh M9 minimal medium to obtain a total volume of 3 mL, before growing overnight in test tubes with agitation. The cells were then inoculated into 4 mL of fresh M9 minimal medium in test tubes containing 0 or 100 μM of cysteine to obtain an initial optical density at 600 nm (OD_600_) of 0.005. Growth (OD_660_) was monitored automatically in a TN-1506 incubator (Advantec Toyo, Tokyo, Japan).

### Quantitative RT-PCR

A 1:100 dilution of an overnight culture of *E. coli* MG1655 grown in M9 minimal medium was inoculated into 25 mL of the same fresh M9 medium in a flask. Cysteine (100 μM) was added after cells reached logarithmic phase (approximately 7 h) with OD_600_ ~0.3. Samples were obtained after 0, 5, and 20 min and they were mixed with RNA Protect Bacteria Reagent (Qiagen, USA), frozen in liquid N_2_, and stored at −80 °C. Total RNA was prepared using an RNeasy Mini Kit (Qiagen, USA). Subsequently, the samples were treated with DNase TURBO DNA-free (Ambion, USA), followed by reverse transcription using an ExScript RT Reagent Kit (Takara Bio, Japan). Using the primers listed in Table 2 and a Power SYBR Green Master Mix (Applied Biosystems, USA), cDNA was quantified by real-time PCR according to the manufacturer’s instructions. Transcript levels were normalized against that of the 16S ribosomal RNA internal standard.

### Fermentative production of cysteine

Each strain was streaked onto an LB plate and grown overnight. Cells were collected using a 10-µL loop, which was passed through 7 cm of bacteria spread, were fully grown on the culture plates, and then inoculated into 2 mL of fermentation medium in test tubes (23 mm internal diameter × 200 mm length). The cells were incubated at 32 °C with agitation until all sugar was consumed, unless stated otherwise. The cysteine level in the culture was quantitatively analyzed according to the method described by Gaitonde (Gaitonde 1967). Before adding the ninhydrin reagent, samples were reduced by incubation with 10 mM dithioerythritol in 10 mM Tris–HCl buffer at pH 8.5 for 10 min. This assay system facilitates the determination of cysteine in its oxidized form (cystine) as well as its condensation product with pyruvate (2-methyl-2,4-thiazolidine carboxylic acid) (Dassler et al. 2000; Wiriyathanawudhiwong et al. 2009). Since these derivatives are easily converted to cysteine via either simple biological or chemical reaction, the assay provides an informative output for the evaluation of ability to produce cysteine as the total cysteine. Cysteine yield was defined as grams of the final amount of the product obtained from 1 g of glucose, which was expressed as a percentage.

## Results

### Screening the genomic library for genes that confer cysteine resistance identified genes involved in cysteine efflux and cysteine degradation

**Fig. 1:**
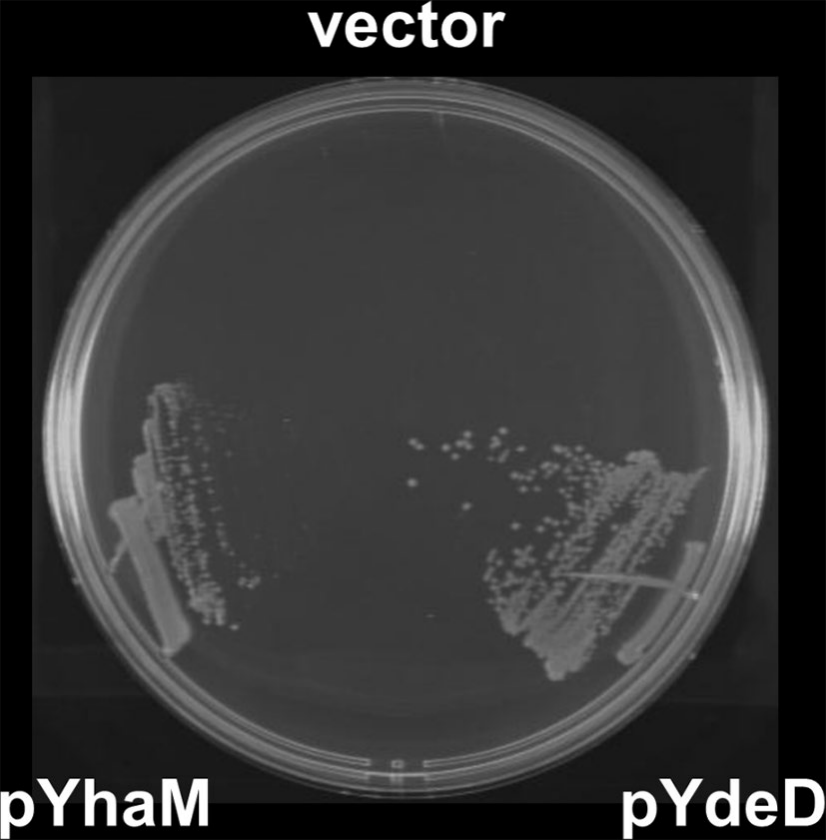
*YhaM* confers cysteine resistance. *E. coli* MG1655 carrying vector (pSTV29), *ydeD* on plasmid (pSTV-ydeD5), and *yhaM* on plasmid (pSTV-yhaM7) were streaked on a M9 plate containing 4 mM cysteine and incubated at 37 °C for 2 days

**Fig. 2:**
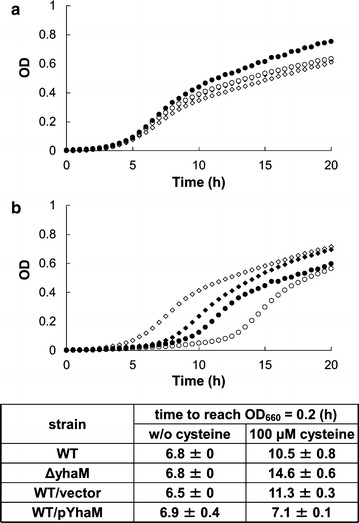
Effects of *yhaM* expression on growth in the presence of cysteine at toxic concentrations. **a** Growth curves for wild-type *E. coli* MG1655 (*closed diamond*), MG1655Δ*yhaM* (*open diamond*), MG1655/vector (pSTV29) (*closed circle*), and MG1655/pYhaM (pSTV-yhaM7) (*open circle*) in the presence of 0 μM (*top*) or 100 μM (*bottom*) of cysteine. Representative growth curves are shown. **b** Culture time (*h*) required for wild-type *E. coli* MG1655, MG1655Δ*yhaM*, MG1655/vector (pSTV29), and MG1655/pYhaM (pSTV-yhaM7) to obtain OD_660_ = 0.2 in the absence (w/o cysteine) or presence of cysteine (100 μM). Values represent the averages based on two independent experiments

To identify *E. coli* genes that confer cysteine resistance when overexpressed, wild-type bacterial strain MG1655 was transformed with a multicopy genomic library of MG1655 and cells were challenged on M9 minimal medium plates supplemented with 2–4 mM cysteine. These conditions showed that the toxicity of cysteine was sufficient to inhibit colony formation for up to 2 days. Colonies were selected after 2 days and the genes that contributed to this phenotype were identified by sequencing the plasmid DNA. In our previous studies, we screened a genomic library of *P. ananatis* using the same system and obtained genes involved in cysteine efflux (*cefA* and *cefB*) and degradation (*ccdA*) (Takumi and Nonaka 2016). The genes obtained were validated as targets for metabolic engineering for cysteine fermentation based on *P. ananatis* either by enhancing the efflux or decreasing the degradation activity, and they were shown to be essential for establishing the overproduction of cysteine (Takumi et al. 2017). Therefore, this screening system is suitable for identifying significant factors. In the present study, screening using *E. coli* identified two loci containing *ydeD*, a cysteine efflux pump involved in the cysteine/cystine shuttle system that protects cells from reactive oxygen species (Dassler et al. 2000; Ohtsu et al. 2010) and *yhaM*, a homolog of the *Methanocaldococcus jannaschii cdsB*, which encodes a cysteine desulfidase that is presumably involved in cysteine degradation (Tchong et al. 2005). Screening conditions were reproduced using *ydeD* and *yhaM* cloned from *E. coli*, each containing 300 bp upstream and 200 bp downstream of the corresponding genes, thereby confirming that the resistance to cysteine was related to these genes (Fig. 1). Figure 2 shows the growth of *E. coli* wild-type strain MG1655 when challenged under conditions containing toxic levels of cysteine (100 μM) in M9 minimal medium. Overexpression of *yhaM* improved growth in these conditions, which was consistent with the typical effects of enhanced cysteine degradation (the lag reduced; see (Takumi and Nonaka 2016) for the growth curve with *P. ananatis ccdA* enhancement). Deletion of *yhaM* had negative effects on growth in the presence of cysteine, thereby suggesting that YhaM is involved in cysteine resistance via degradation. This screening system and phenotypic characterization of growth in the presence of cysteine identified factors related to efflux and degradation, the regulation of which may be effective for cysteine production (Takumi and Nonaka 2016). Indeed, one of the genes identified by screening, *ydeD*, has been studied and characterized extensively as a cysteine efflux pump with a central role in cysteine production by *E. coli* (Dassler et al. 2000). Therefore, we focused on *yhaM* to characterize its possible application in cysteine production.

### *yhaM* is cysteine inducible

**Fig. 3:**
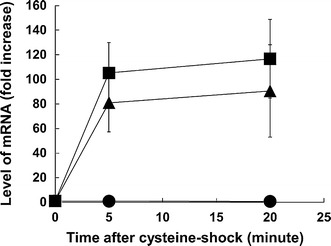
Induction of *yha*O and *yhaM* transcription by cysteine. Time course of the relative *yhaO* (*square*), *yhaM* (*triangle*), and *metC* (*circle*) mRNA levels after the addition of cysteine (cysteine-shock). Relative mRNA levels compared with the mRNA level before cysteine addition are shown. Values represent the averages of the results based on three independent experiments and the *error bar* represents one standard deviation

To explore the physiological significance of YhaM for the detoxification of cysteine in *E. coli*, we performed transcriptional analysis to probe its expression in response to cysteine shock (addition of exogenous cysteine) using quantitative RT-PCR. Figure 3 shows the mRNA levels of *yhaO* and *yhaM*, with *metC* as a negative control. *yhaO* is located upstream of *yhaM* and they are predicted to be organized in an operon (regulon DB; http://regulondb.ccg.unam.mx/). Our findings demonstrated that *yhaM* and *yhaO* expression was significantly induced after cysteine shock. The response was rapid (less than 5 min) and strong (ca 80- and 100-fold increases for *yhaM* and *yhaO*, respectively), which is consistent with an idea that YhaM is responsive to cysteine (see “Discussion” for the significance of the speed and magnitude of the induction). The results support the hypothesis that YhaM acts in cysteine degradation, which is associated with cysteine detoxification in *E. coli* [see “Discussion” for the function of YhaM deduced from this study as well as previous studies (Shimada et al. 2016; Takumi et al. 2017; Tchong et al. 2005)].

### YhaM modulates cysteine production

YhaM serves as a primary degradation enzyme for cysteine detoxification via cysteine-mediated induction in *E. coli*, so it may affect cysteine production and it could be an important target for the genetic engineering of cysteine-producing strains of *E. coli*. To explore this possibility, we deleted and overexpressed the *yhaM* gene in a model cysteine-producing strain based on *E. coli* MG1655 and performed a fermentation test to evaluate its production of cysteine. In *E. coli*, cysteine synthesis is regulated primarily by SAT, which is encoded by *cysE*, and it is subject to feedback inhibition by cysteine. Cells that express a feedback-insensitive mutant of SAT produce traces of cysteine and they can be exploited as a simple cysteine-producing strain (Yamada et al. 2006). Efflux of the synthesized cysteine from the cells is the production bottleneck for this model strain, where only traces of the cysteine produced accumulate in the production medium and most of the product is decomposed in the cells by degradation enzymes. Indeed, the production of cysteine in this model strain based on *P. ananatis* (i.e., *P. ananatis* carrying the mutant SAT), a close relative and an alternative promising host to *E. coli*, was increased by deleting the major CD, CcdA (Takumi et al. 2017). Therefore, *E. coli* carrying the mutant SAT is a suitable model for evaluating the effects of YhaM. Therefore, we employed the *E. coli* wild-type strain MG1655 harboring the plasmid pMIV-cysE5 carrying a *cysE5* gene (Kai et al. 2006), which encodes a feedback-insensitive mutant of SAT, to determine whether *yhaM* expression can affect cysteine production. Tables 3 and 4 show the fermentative production of cysteine under conditions where *yhaM* was overexpressed from a multicopy plasmid and where it was deleted, respectively. Production of cysteine decreased significantly when *yhaM* was overexpressed (Table 3) whereas it increased drastically when *yhaM* was deleted (Table 4), which suggests that YhaM was in an active state during cysteine production when it was present. These findings suggest that *yhaM* may be a promising target for genetic engineering to obtain high-performance cysteine-producing strains at industrial production levels.

**Table 3:** Effects of *yhaM* overexpression on the production of cysteine

Strain	Plasmid	Cysteine (mg L^−1^)	Yield (%)	OD_660_
MG1655 (WT)	pMIV-cysE5, pACYC177	74.6 ± 7.0	0.187 ± 0.018	21.7 ± 0.8
	pMIV-cysE5, pACYC-yhaM1	14.9 ± 1.1	0.037 ± 0.003	22.0 ± 0.8

Values represent the averages based on five independent experiments and the error value represents one standard deviation. To retain the plasmids, 20 μg mL^−1^ kanamycin and 25 μg mL^−1^ chloramphenicol were added. Cultivation was terminated at 21–24 h after inoculation when all the sugar (40 g L^−1^) had been consumed

**Table 4:** Effects of *yhaM* deletion on the production of cysteine

Strain	Plasmid	Cysteine (mg L^−1^)	Yield (%)	OD_660_
MG1655 (WT)	pMIV-cysE5	64.6 ± 4.5	0.164 ± 0.011	28.6 ± 0.8
MG1655 Δ*yhaM*	pMIV-cysE5	316.2 ± 74.4	0.810 ± 0.195	29.4 ± 0.9

Values represent the averages based on four independent experiments and the error value represents one standard deviation. To retain the plasmids, 25 μg mL^−1^ chloramphenicol was added. Cultivation was terminated at 15–17 h after inoculation when >95% of the total sugar (>38 g L^−1^) had been consumed

## Discussion

YhaM was originally characterized as a cysteine desulfidase in *M. jannaschii* (Tchong et al. 2005). Recently, Ishihama et al. characterized YhaM in *E. coli*, where they demonstrated that *yhaM* encodes a cysteine-inducible hydrogen sulfide-forming cysteine degradative enzyme, which is involved in the detoxification of cysteine (Shimada et al. 2016). These findings are consistent with our claim that YhaM may catalyze the main degradative activity of cysteine in *E. coli*, thus making it one of the key factors required for establishing efficient fermentative production of cysteine. Our screening study focused on cysteine resistance, characterization of a direct cysteine stimulon with a rapid and strong response, and its applications to cysteine fermentation, where these approaches provided important insights into the crucial factors that affect cysteine fermentation using *E. coli*.

Multiple enzymes have CD activities in *E. coli*, but most of them are involved in other physiological functions via the distinct enzymatic activity of CD, so the significance of their activity and roles in cysteine degradation remain unclear. Based on our findings as well as findings from recent studies, we propose that *yhaM* encodes the major cysteine degradation enzyme in *E. coli*. Several key findings support this hypothesis. First, our screening approach is well established where it identified the major CD called CcdA and its cysteine-responsive regulator CcdR in *P. ananatis* (Takumi and Nonaka 2016). It is important to note that none of the known CDs in *E. coli* were identified by screening, which suggests that their contributions as cysteine scavengers might be insignificant. Second, *yhaM* expression was cysteine-inducible. In *E. coli*, *tnaA* was reported to be cysteine-responsive (Awano et al. 2005), but our cysteine-shock experiment failed to detect cysteine-mediated induction (data not shown), which indicates that *tnaA* is not a direct cysteine stimulon. Findings from a recent study are consistent with our finding concerning the cysteine-mediated induction of *yhaM*, where the *yhaM* mRNA levels were upregulated when cells were cultivated in the presence of cysteine (Shimada et al. 2016). The present study investigated the properties based on kinetics, where we showed that the induction of *yhaM* expression in response to cysteine was significant in terms of both its speed and magnitude, which strongly suggests that the induction was direct. Third, *yhaM* is organized in an operon with *yhaO*, which encodes a cysteine transporter (Shimada et al. 2016). Severity of cysteine toxicity in the periplasm was recently demonstrated (Ohtsu et al. 2010, 2015). Therefore, it is reasonable that cells control the main decomposer as well as a specific transporter at the transcriptional level, which may explain why *yhaO* has *yhaM* as a partner among the many CDs. Finally, modulating the expression of *yhaM* via deletion or overexpression drastically affected the ability of the cells to produce cysteine. These phenotypic features are consistent with our previous findings in *P. ananatis* based on its main CD encoded by *ccdA* (Takumi and Nonaka 2016).

**Fig. 4:**
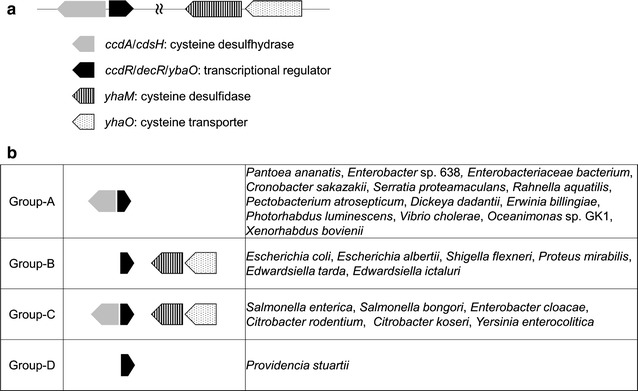
Conservation and variation of cysteine degradation systems in Enterobacteriaceae bacteria. **a** Genomic context of the genes in the bacterial cysteine degradation systems conserved in bacteria. **b** Comparison of the genomic contexts of the bacterial cysteine degradation systems among genomes of Enterobacteriaceae bacteria. Representative examples of bacterial genomes are shown. Note that *Xenorhabdus bovienii* contains *yhaO*, and some *Escherichia coli* strains (e.g., CFT073) and *Salmonella enterica* strains (e.g., CT18) lack *yhaO*; thus, they represent subgroups. Data were obtained using the web resource STRING (http://www.string-db.org/)

Recently, the induction of the YhaM by cysteine was shown to be mediated by a transcriptional regulator YbaO (Shimada et al. 2016). Interestingly, the major cysteine degradation enzyme in *P. ananatis*, CcdA (and its *S. enterica* ortholog, CdsH), is also cysteine-inducible, where induction is mediated by the YbaO ortholog of *P. ananatis*, CcdR (*S. enterica* ortholog, DecR) (Oguri et al. 2012; Takumi and Nonaka 2016). Therefore, these two bacterial cysteine degradation systems (i.e., YhaM and CcdA/CdsH) that catalyze the same cysteine-decomposing reaction, share the same transcriptional regulator (i.e., YbaO/CcdR/DecR). However, they share poor sequence identity, and thus they are classified as different enzymes (CD for CcdA/CdsH and cysteine desulfidase for YhaM). In *P. ananatis* (and *S. enterica*), *ccdA* is located adjacent to *ccdR* on the genome and they form a gene cluster. On the other hand, in *E. coli*, *ccdA* is missing from the position adjacent to *ybaO* and the *yhaOM* operon is instead located at another locus in the genome. These findings suggest an interrelationship between two distinct cysteine degradation systems via a common transcriptional regulator CcdR/DecR/YbaO among bacteria, which motivated us to explore the conservation and variation of these systems among bacteria (see Fig. 4a for a summary of the main components). YhaM-YhaO is conserved in a limited number of bacterial species. It is mainly conserved among some γ-Proteobacteria members, particularly Enterobacteriaceae. Interestingly, this conservation varies even within a single species, e.g., both *yhaO* and *yhaM* are present in *E. col*i K-12 and O157:H1, but only *yhaM* is present in CFT073. Likewise, *S. enterica* CT18 contains *yhaOM*, whereas LT2 contains only *yhaM*. Moreover, conservation of the CcdR-CcdA/DecR-CdsH system is also limited to γ-Proteobacteria, particularly Enterobacteriaceae. We queried the STRING database (Snel et al. 2000) to obtain an overview of the conservation and variation in the arrangement of genes among the bacteria in Enterobacteriaceae and identified seven groups based on the genome context (Fig. 4b). Group-A and group-B comprise the *P. ananatis*-type and the *E. coli*-type gene arrangements, respectively. Interestingly, a group of bacteria carry the full set of genes, i.e., group-C. Group-D bacteria contain the regulator *ybaO/ccdR*, which is missing with its counterpart *ccdA* and *yhaOM*. Thus, even in the same Enterobacteriaceae families, variations in the bacterial conservation of this system presumably reflect their complex requirements under specific environmental conditions, which have developed by evolution. Understanding the entire degradation systems and determining the necessary genetic modifications required for their targeting are crucial for improving the bacterial fermentation-based production of cysteine.

Owing to the toxicity of cysteine, cells are equipped with complex mechanisms for its degradative regulation. Therefore, managing cellular degradation activity is an essential approach for establishing efficient fermentative production of cysteine. Having clarified the core decomposers of cysteine in *E. coli*, the next step to facilitate the regulation of cysteine degradation will be fine-tuning each of the degradative genes in combination with the cellular efflux capacity. In *E. coli*, except for YhaM, all cysteine degradation enzymes have physiological functions other than cysteine decomposition; simply deleting the CD genes might perturb important physiological functions, thereby negatively affecting the process of fermentation. Therefore, carefully selecting the target genes and fine-tuning their expression levels will be important for genetic engineering. In particular, *yhaM* might be the main target among the many genes involved in cysteine degradation because it is specific to cysteine and it may have less negative impacts on various fermentation profiles. Indeed, according to our findings, the deletion of *yhaM* showed no negative effects on the OD, cultivation time, and other fermentation parameters.

Stepwise genetic engineering of cysteine-producing strains has been demonstrated in *P. ananatis*, where the core factors related to cysteine production were modulated in combination, including feedback-insensitive mutant alleles of SAT and 3-PGDH, efflux pumps, and the major CD (Takumi et al. 2017). Furthermore, rate-limiting steps in the cysteine biosynthetic pathway were enhanced in addition to these core factors. Most of the core factors are currently known following the identification of the major and minor CDs in *E. coli*. Therefore, the next challenge for *E. coli*-based cysteine production will be stepwise genetic engineering by targeting and fine-tuning the core factors in combination with the rate-limiting biosynthetic genes.

## Abbreviations

SAT: serine acetyltransferase
3-PGDH: 3-phosphoglycerate dehydrogenase
CD: cysteine desulfhydrase
LB: Luria–Bertani
OD: optical density
RT-PCR: real time polymerase chain reaction
WT: wild type
